# Climate Change and Vector-Borne Disease Transmission: The Role of Insect Behavioral and Physiological Adaptations

**DOI:** 10.1093/iob/obaf011

**Published:** 2025-03-19

**Authors:** E Abbasi

**Affiliations:** Faculty of Public Health, Department of Biology and Vector Control, Shiraz University of Medical Sciences, Zand Street, JGHF+XFG, Shiraz 3761833650, Fars Province, Iran; Department of Medical Entomology and Vector Control, School of Health, Shiraz University of Medical Sciences, Shiraz 3761833650, Iran

## Abstract

Climate change is profoundly reshaping the behavior, physiology, and distribution of insect vectors, with significant implications for vector-borne disease transmission. Rising temperatures, shifting precipitation patterns, and extreme weather events are driving behavioral adaptations such as altered host-seeking patterns, modified resting site preferences, and extended seasonal activity. Concurrently, vectors exhibit physiological plasticity, including enhanced thermal tolerance, desiccation resistance, and accelerated reproductive cycles, which contribute to increased survival and vector competence. This review synthesizes current research on climate-driven adaptations in major disease vectors, focusing on their epidemiological consequences and implications for public health interventions. A systematic literature review was conducted using major scientific databases to assess the impact of climate change on insect vector adaptation. Studies examining temperature-induced behavioral shifts, physiological modifications, and changes in vector competence were analyzed to identify emerging trends and knowledge gaps. Findings indicate that climate-driven vector adaptations are increasing the efficiency of disease transmission, enabling the geographic expansion of vector populations and prolonging transmission seasons. These changes challenge existing vector control strategies, necessitating innovative approaches such as genetic engineering, microbiome-based interventions, and climate-informed surveillance systems. Given the accelerating impact of climate change, there is an urgent need for adaptive, evidence-based control strategies to mitigate the growing threat of vector-borne diseases and enhance global health resilience.

## Synopsis (German)

Der Klimawandel verändert tiefgreifend das Verhalten, die Physiologie und die Verbreitung von Insektenvektoren, was erhebliche Auswirkungen auf die Übertragung vektorübertragener Krankheiten hat. Steigende Temperaturen, veränderte Niederschlagsmuster und extreme Wetterereignisse führen zu Verhaltensanpassungen wie veränderten Wirtsuchmustern, modifizierten Ruheplatzpräferenzen und verlängerter saisonaler Aktivität. Gleichzeitig zeigen Vektoren physiologische Plastizität, darunter eine erhöhte Hitzetoleranz, Resistenz gegen Austrocknung und beschleunigte Fortpflanzungszyklen, was zu einer höheren Überlebensrate und Vektorkompetenz beiträgt.Diese Übersichtsarbeit fasst aktuelle Forschungsergebnisse zu klimabedingten Anpassungen wichtiger Krankheitsvektoren zusammen, mit Schwerpunkt auf ihren epidemiologischen Folgen und Implikationen für öffentliche Gesundheitsmaßnahmen. Eine systematische Literaturrecherche wurde in führenden wissenschaftlichen Datenbanken durchgeführt, um die Auswirkungen des Klimawandels auf die Anpassung von Insektenvektoren zu bewerten. Studien, die temperaturinduzierte Verhaltensänderungen, physiologische Modifikationen und Veränderungen der Vektorkompetenz untersuchten, wurden analysiert, um aufkommende Trends und Forschungslücken zu identifizieren.Die Ergebnisse zeigen, dass klimabedingte Anpassungen von Vektoren die Effizienz der Krankheitsübertragung erhöhen, die geografische Ausbreitung von Vektorpopulationen begünstigen und die Übertragungszeiten verlängern. Diese Veränderungen stellen bestehende Vektorkontrollstrategien vor große Herausforderungen und erfordern innovative Ansätze wie genetische Modifikationen, mikrobiombasierte Interventionen und klimainformierte Überwachungssysteme. Angesichts der zunehmenden Auswirkungen des Klimawandels besteht ein dringender Bedarf an adaptiven, evidenzbasierten Kontrollstrategien, um die wachsende Bedrohung durch vektorübertragene Krankheiten zu mindern und die globale Gesundheitsresilienz zu stärken.

## Resumen (Spanish)

El cambio climático está remodelando profundamente el comportamiento, la fisiología y la distribución de los insectos vectores, con importantes implicaciones para la transmisión de enfermedades transmitidas por vectores. El aumento de las temperaturas, los cambios en los patrones de precipitación y los eventos climáticos extremos están impulsando adaptaciones conductuales, como modificaciones en la búsqueda de hospedadores, cambios en las preferencias de los sitios de reposo y una actividad estacional prolongada. Paralelamente, los vectores exhiben plasticidad fisiológica, incluyendo una mayor tolerancia térmica, resistencia a la desecación y ciclos reproductivos acelerados, lo que contribuye a una mayor supervivencia y competencia vectorial. Esta revisión sintetiza la investigación actual sobre las adaptaciones impulsadas por el clima

en los principales vectores de enfermedades, centrándose en sus consecuencias epidemiológicas y sus implicaciones para las intervenciones en salud pública. Se realizó una revisión sistemática de la literatura utilizando las principales bases de datos científicas para evaluar el impacto del cambio climático en la adaptación de los insectos vectores. Se analizaron estudios que examinan cambios conductuales inducidos por la temperatura, modificaciones fisiológicas y alteraciones en la competencia vectorial con el fin de identificar tendencias emergentes y lagunas de conocimiento. Los hallazgos indican que las adaptaciones de los vectores impulsadas por el cambio climático están aumentando la eficiencia de la transmisión de enfermedades, permitiendo la expansión geográfica de las poblaciones de vectores y prolongando las temporadas de transmisión. Estos cambios desafían las estrategias de control de vectores existentes, lo que hace necesaria la adopción de enfoques innovadores, como la ingeniería genética, intervenciones basadas en el microbioma y sistemas de vigilancia informados por el clima. Dado el impacto acelerado del cambio climático, es urgente desarrollar estrategias de control adaptativas y basadas en evidencia para mitigar la creciente amenaza de las enfermedades transmitidas por vectores y fortalecer la resiliencia sanitaria global.

## Synopsis (Italian)

Il cambiamento climatico sta rimodellando profondamente il comportamento, la fisiologia e la distribuzione degli insetti vettori, con importanti implicazioni per la trasmissione delle malattie trasmesse da vettori. L'aumento delle temperature, le variazioni nei modelli di precipitazione e gli eventi meteorologici estremi stanno determinando adattamenti comportamentali, come cambiamenti nei modelli di ricerca dell'ospite, modifiche nelle preferenze dei siti di riposo e un'attività stagionale prolungata. Contemporaneamente, i vettori mostrano una plasticità fisiologica, tra cui una maggiore tolleranza termica, resistenza alla disidratazione e cicli riproduttivi accelerati, contribuendo a una maggiore sopravvivenza e competenza vettoriale. Questa revisione sintetizza le ricerche attuali sulle adattamenti dei principali vettori di malattie indotte dal cambiamento climatico, concentrandosi sulle conseguenze epidemiologiche e sulle implicazioni per le strategie di sanità pubblica. È stata condotta una revisione sistematica della letteratura utilizzando le principali banche dati scientifiche per valutare l'impatto del cambiamento climatico sull'adattamento degli insetti vettori. Sono stati analizzati studi che esaminano le variazioni comportamentali indotte dalla temperatura, le modificazioni fisiologiche e i cambiamenti nella competenza vettoriale al fine di identificare tendenze emergenti e lacune di conoscenza. I risultati indicano che gli adattamenti dei vettori determinati dal cambiamento climatico stanno aumentando l'efficienza della trasmissione delle malattie, consentendo l'espansione geografica delle popolazioni vettoriali e prolungando le stagioni di trasmissione. Questi cambiamenti sfidano le attuali strategie di controllo dei vettori, rendendo necessaria l'adozione di approcci innovativi come l'ingegneria genetica, le interventi basati sul microbioma e i sistemi di sorveglianza informati dal clima. Considerando l'impatto accelerato del cambiamento climatico, è urgente sviluppare strategie di controllo adattive e basate sull'evidenza per mitigare la crescente minaccia delle malattie trasmesse da vettori e rafforzare la resilienza sanitaria globale.

## Synopsis (Arabic)

يُعيد التغير المناخي تشكيل سلوك الحشرات الناقلة للأمراض وفسيولوجيتها وتوزيعها بشكل عميق، مما يترتب عليه آثار كبيرة على انتقال الأمراض المنقولة بالنواقل. تؤدي ارتفاع درجات الحرارة، وتغير أنماط الهطول، والظواهر الجوية المتطرفة إلى تغييرات سلوكية مثل تعديل أنماط البحث عن العائل، وتفضيلات مواقع الاستراحة، وزيادة النشاط الموسمي. بالتزامن مع ذلك، تظهر النواقل مرونة فسيولوجية تشمل زيادة تحمل الحرارة، ومقاومة الجفاف، وتسريع دورات التكاثر، مما يسهم في زيادة معدلات البقاء والكفاءة الناقلة. تستعرض هذه المراجعة الأبحاث الحالية حول التكيفات التي تحركها التغيرات المناخية في النواقل الرئيسية للأمراض، مع التركيز على العواقب الوبائية والتداعيات على التدخلات الصحية العامة. تم إجراء مراجعة منهجية للأدبيات العلمية باستخدام قواعد البيانات العلمية الكبرى لتقييم تأثير التغير المناخي على تكيف الحشرات الناقلة للأمراض. تم تحليل الدراسات التي تفحص التغيرات السلوكية الناجمة عن درجة الحرارة، والتعديلات الفسيولوجية، والتغيرات في كفاءة النقل، بهدف تحديد الاتجاهات الناشئة والفجوات المعرفية. تشير النتائج إلى أن التكيفات المناخية للنواقل تزيد من كفاءة انتقال الأمراض، مما يسمح بالتوسع الجغرافي لمجموعات النواقل وإطالة مواسم الانتقال. تشكل هذه التغيرات تحديًا لاستراتيجيات مكافحة النواقل الحالية، مما يستلزم اعتماد أساليب مبتكرة مثل الهندسة الوراثية، والتدخلات القائمة على الميكروبيوم، وأنظمة المراقبة المستندة إلى المناخ. نظرًا للتأثير المتسارع للتغير المناخي، هناك حاجة ملحة إلى استراتيجيات تكيفية قائمة على الأدلة للحد من التهديد المتزايد للأمراض المنقولة بالنواقل وتعزيز مرونة الصحة العالمية.

الكلمات المفتاحية

## Synopsis (Persian)

تغییرات اقلیمی به‌طور چشمگیری رفتار، فیزیولوژی و پراکنش ناقلین حشرات را دگرگون کرده و پیامدهای مهمی برای انتقال بیماری‌های منتقله توسط ناقلین به همراه دارد. افزایش دما، تغییر الگوهای بارندگی و رخدادهای شدید جوی باعث سازگاری‌های رفتاری مانند تغییر در الگوهای جستجوی میزبان، اصلاح ترجیحات زیستگاه‌های استراحت و افزایش فعالیت فصلی ناقلین شده است. هم‌زمان، ناقلین انعطاف‌پذیری فیزیولوژیکی قابل‌توجهی از خود نشان می‌دهند، از جمله افزایش تحمل حرارتی، مقاومت در برابر خشکی و چرخه‌های تولیدمثلی سریع‌تر که به بقای بیشتر و افزایش کارایی آن‌ها در انتقال بیماری‌ها کمک می‌کند. این مطالعه مروری به بررسی تحقیقات اخیر در مورد سازگاری‌های ناقلین اصلی بیماری‌ها در پاسخ به تغییرات اقلیمی پرداخته و پیامدهای اپیدمیولوژیک و مداخلات سلامت عمومی مرتبط را مورد تحلیل قرار داده است.

یک مرور سیستماتیک از مطالعات منتشرشده در پایگاه‌های داده علمی معتبر برای ارزیابی اثر تغییرات اقلیمی بر سازگاری ناقلین حشرات انجام شد. در این مطالعه، تحقیقات مرتبط با تغییرات رفتاری ناشی از دما، اصلاحات فیزیولوژیکی و تغییرات در کارایی انتقال بیماری توسط ناقلین مورد تجزیه‌وتحلیل قرار گرفت تا روندهای نوظهور و شکاف‌های دانشی شناسایی شوند. یافته‌ها نشان می‌دهند که سازگاری‌های اقلیمی ناقلین باعث افزایش کارایی انتقال بیماری، گسترش جغرافیایی جمعیت‌های ناقل و طولانی‌تر شدن فصول انتقال بیماری می‌شود. این تغییرات راهبردهای کنونی کنترل ناقلین را به چالش کشیده و نیازمند به‌کارگیری رویکردهای نوآورانه‌ای مانند مهندسی ژنتیک، مداخلات مبتنی بر میکروبیوم و سیستم‌های پایش مبتنی بر تغییرات اقلیمی است. با توجه به تأثیرات فزاینده تغییرات اقلیمی، تدوین راهبردهای کنترلی سازگار و مبتنی بر شواهد برای کاهش تهدید روزافزون بیماری‌های منتقله توسط ناقلین و تقویت تاب‌آوری جهانی در برابر این بیماری‌ها ضروری است.

واژگان کلیدی

## Introduction

Vector-borne diseases (VBDs) constitute a major public health challenge, disproportionately affecting populations in tropical and subtropical regions. These diseases, transmitted primarily by hematophagous arthropods such as mosquitoes, ticks, and sandflies, are responsible for a significant global disease burden, contributing to high morbidity and mortality rates (Blum et al. 2018). Among the most notorious VBDs are malaria, dengue, chikungunya, Zika virus, leishmaniasis, and Lyme disease, all of which are influenced by environmental factors that shape the dynamics of pathogen transmission. In recent decades, climate change has emerged as one of the most critical drivers of shifts in the epidemiology of VBDs, altering the geographic distribution, seasonality, and vector competence of insect populations (Ryan et al. 2019). As global temperatures rise, precipitation patterns shift, and extreme weather events become more frequent, the ecological interactions between vectors, pathogens, and hosts are being fundamentally reshaped, raising concerns about the emergence and re-emergence of VBDs in previously unaffected regions (Weaver et al. 2018).

The influence of climate change on VBD transmission is multifaceted, encompassing both direct and indirect effects on insect vectors. Directly, rising temperatures accelerate vector development, shorten extrinsic incubation periods (EIPs) of pathogens, and enhance vector biting rates, thereby increasing transmission potential. Indirectly, climate-driven changes in habitat suitability, host availability, and competition among species influence vector population dynamics and pathogen persistence. However, a critical yet often overlooked aspect of climate change-induced shifts in disease transmission lies in the behavioral, physiological, and insecticide resistance adaptations of insect vectors, which collectively impact the effectiveness of traditional vector control measures (Murdock et al. 2016; Caminade et al. 2019). Vectors, particularly mosquitoes and ticks, exhibit remarkable phenotypic plasticity, enabling them to respond to environmental fluctuations through modifications in behavior (e.g., host-seeking, resting site selection, and feeding times) and physiological processes (e.g., thermal tolerance, desiccation resistance, and immune responses). These adaptations not only determine the survival and fitness of vector populations under changing climatic conditions but also mediate pathogen acquisition, retention, and transmission efficiency, ultimately shaping epidemiological outcomes (Rocklöv et al. 2016).

Recent research has provided compelling evidence that vectors adapt to climate-induced stressors through genetic, epigenetic, and behavioral modifications, underscoring the need for a deeper understanding of these adaptive mechanisms (Doré et al. 2021). For instance, shifts in host preference and altered feeding behaviors have been observed in *Aedes aegypti* mosquitoes in response to urbanization and temperature extremes, influencing their role as primary vectors of arboviral diseases. Similarly, ticks such as *Ixodes scapularis*, the vector of Lyme disease, exhibit altered questing behaviors and extended seasonal activity periods as a result of warming temperatures, facilitating the northward expansion of tick-borne infections. In addition, physiological plasticity in response to thermal stress has been documented in several vector species, with increased heat tolerance linked to enhanced survival and reproductive success under changing climatic conditions. Despite these insights, gaps remain in our understanding of the molecular, physiological, and ecological underpinnings of vector adaptation, particularly in the context of disease transmission risk (Moesker et al. 2013; Rohr et al. 2019).

This review aims to provide a comprehensive synthesis of the behavioral and physiological adaptations of insect vectors in response to climate change and their implications for VBD transmission (Carlson et al. 2022). By integrating findings from entomology, epidemiology, and climate science, we will explore how vectors adjust their life history traits and host-seeking behaviors to persist in increasingly unpredictable environments. Furthermore, we will highlight the potential consequences of these adaptations for disease surveillance, vector control strategies, and global health preparedness (Liu-Helmersson et al. 2014). Given the accelerating pace of climate change and its profound impact on vector ecology, understanding these adaptive responses is critical for predicting future disease outbreaks and developing effective mitigation strategies (Rueda et al. 1990).

## Materials and methods

To comprehensively examine the behavioral and physiological adaptations of insect vectors to climate change and their implications for VBD transmission (Parham and Michael 2010; Mordecai et al. 2019), this review employs a systematic and integrative approach. Given the interdisciplinary nature of this topic, which spans entomology, epidemiology, climate science, and vector ecology, a rigorous methodology was designed to ensure the inclusion of high-quality, peer-reviewed studies from diverse yet relevant scientific domains. This section details the methodological framework employed in conducting the literature search, data extraction, synthesis, and analysis (Rocklöv and Dubrow 2020).

### Literature search strategy

A systematic literature search was conducted following the Preferred Reporting Items for Systematic Reviews and Meta-Analyses (PRISMA) guidelines, ensuring a transparent, reproducible, and comprehensive approach to identifying relevant studies (Moher et al. 2009). Multiple electronic databases, including Web of Science, PubMed, Scopus, and Google Scholar, were systematically searched to retrieve original research articles, reviews, and meta-analyses published between 2000 and 2024. The selection of this timeframe reflects the period during which climate change impacts on vector ecology have been extensively studied, alongside advancements in genomic and behavioral research. The search terms were carefully curated to capture studies at the intersection of climate change, vector behavior, vector physiology, and disease transmission dynamics. Keywords and Boolean operators included: (“vector-borne diseases” OR “mosquito-borne diseases” OR “tick-borne diseases” OR “arboviruses” OR “malaria” OR “dengue” OR “leishmaniasis” OR “Lyme disease”) AND (“climate change” OR “global warming” OR “temperature increase” OR “precipitation changes” OR “environmental change” OR “extreme weather events”) AND (“vector adaptation” OR “physiological plasticity” OR “behavioral modification” OR “thermal tolerance” OR “host-seeking behavior” OR “feeding behavior” OR “reproductive adaptation” OR “vector competence”) (Campbell-Lendrum et al. 2015). Additionally, backward and forward citation tracking was performed to identify relevant studies that may not have been captured in the initial database search. Gray literature, including reports from the World Health Organization, Intergovernmental Panel on Climate Change, and Centers for Disease Control and Prevention, was also reviewed to incorporate policy-relevant insights (Patz et al. 2005).

### Inclusion and exclusion criteria

To ensure the relevance and rigor of selected studies, predefined inclusion and exclusion criteria were applied. Studies were included if they investigated climate change-induced behavioral or physiological adaptations in insect vectors (e.g., mosquitoes, ticks, sandflies, and blackflies); provided empirical data on changes in vector ecology, biting rates, host-seeking behavior, thermal tolerance, reproductive success, or immune responses under varying environmental conditions; employed robust experimental, observational, or modeling approaches to analyze vector responses to climate change; and were published in English in peer-reviewed journals or reputable institutional reports (Caminade et al. 2017). Studies were excluded if they focused exclusively on vector control interventions without considering natural vector adaptations; addressed climate change and disease transmission without examining vector behavioral or physiological mechanisms; lacked clear methodological descriptions; or relied solely on theoretical speculations without empirical validation (Kilpatrick and Randolph 2012).

### Data extraction and synthesis

For all selected studies, key information was systematically extracted, including vector species studied, geographic location, climate change variables assessed (e.g., temperature, humidity, and precipitation), behavioral or physiological traits examined, methodological approaches used, and major findings. The extracted data were categorized into thematic areas to facilitate synthesis and comparison (Ryan et al. 2019). These thematic areas included examining changes in biting activity, resting site selection, and host preference under elevated temperatures (temperature-driven modifications in vector behavior); evaluating shifts in heat resistance, metabolic adjustments, and immune responses under climate stress (physiological plasticity and thermal tolerance); assessing alterations in EIPs, pathogen susceptibility, and reproductive dynamics in response to climate change (vector competence and disease transmission potential); and documenting range expansions, changes in vector abundance, and seasonal variations in transmission patterns (geographic and seasonal shifts in vector distribution) (Brady et al. 2014). A narrative synthesis approach was employed to integrate findings across studies, highlighting both consistencies and discrepancies in reported vector responses. Where available, meta-analytic techniques were considered to quantify effect sizes related to specific behavioral or physiological traits under varying climatic conditions (Harrington et al. 2005).

### Methodological rigor and bias assessment

To ensure the reliability of synthesized evidence, methodological rigor was critically evaluated using established quality assessment tools, including the Newcastle-Ottawa Scale for observational studies and the risk of bias (RoB) assessment framework for experimental research (Egger et al. 1997; Wells et al. 2000). Particular attention was given to sample size adequacy and study design robustness; potential confounders, such as land use changes, human interventions, and coinfections, which could influence reported vector adaptations; and statistical methodologies employed to model climate-driven vector responses. Additionally, publication bias was assessed using funnel plot asymmetry and Egger's test, particularly in cases where multiple studies examined similar adaptation mechanisms. Studies with high risk of bias or insufficient methodological clarity were excluded from quantitative synthesis but referenced in discussion sections to acknowledge potential uncertainties (Gates and Woolhouse 2015).

### Limitations and scope considerations

While this review aims to provide a comprehensive synthesis of vector adaptation mechanisms in response to climate change, certain limitations must be acknowledged. First, regional variations in vector response patterns may be influenced by local climate trends, host availability, and anthropogenic factors, which are difficult to standardize across studies. Second, the complex interplay between genetic and environmental determinants of adaptation is still an evolving field, necessitating further genomic and experimental research. Finally, modeling studies predicting future vector distributions remain inherently uncertain due to the stochastic nature of climate projections and pathogen evolution dynamics (Gould and Higgs 2009; Lambrechts et al. 2011). Despite these challenges, this review provides a robust and evidence-based synthesis of climate-induced vector adaptations, offering valuable insights for epidemiologists, entomologists, and public health policymakers. By systematically integrating behavioral, physiological, and ecological perspectives, this study aims to advance our understanding of how insect vectors are evolving in response to climate change and the implications for future disease emergence and control strategies (Reisen 2010).

## Results

The synthesis of existing literature revealed substantial evidence that climate change is profoundly altering the behavioral and physiological traits of insect vectors, thereby reshaping VBD transmission dynamics worldwide (Rocklöv and Dubrow 2020). The findings were categorized into four major themes: (1) temperature-driven modifications in vector behavior, (2) physiological plasticity and thermal tolerance, (3) vector competence and disease transmission potential, and (4) geographic and seasonal shifts in vector distribution. Each of these aspects provides critical insight into how insect vectors are adapting to changing environmental conditions and how these adaptations influence disease risk (IPOC Change 2007; Nova et al. 2019). For further details, please refer to the supplementary materials of the manuscript.

A growing body of evidence suggests that rising temperatures significantly influence the behavior of insect vectors, particularly in terms of biting activity, host-seeking strategies, and resting site selection. Studies on *A. aegypti* and *Anopheles* spp have demonstrated that elevated temperatures not only increase biting frequency but also alter the timing and intensity of blood-feeding behaviors (Murdock et al. 2016). Several experimental studies indicate that mosquitoes in warmer climates exhibit a shift from nocturnal to crepuscular or even diurnal feeding activity, which has profound implications for disease transmission. The traditional assumption that *Anopheles* mosquitoes primarily bite at night is being challenged by emerging evidence showing that increasing nighttime temperatures drive a shift toward earlier evening or dawn feeding peaks, particularly in urban environments (Brady et al. 2014). Furthermore, host preference has been shown to change in response to rising temperatures. Some studies report that *Aedes* and *Culex* mosquitoes exhibit an increased preference for human hosts in warmer conditions, potentially driven by metabolic demands requiring higher blood meal intake. This shift enhances vector–human contact rates, thereby intensifying disease transmission risks. Additionally, changes in resting site selection have been noted, with vectors increasingly seeking cooler microhabitats in response to heat stress, thereby influencing their exposure to insecticide-treated surfaces and altering control efficacy (Carrington et al. 2013).

Climate change is also driving significant modifications in the physiological responses of insect vectors, particularly in their thermal tolerance, metabolic adjustments, and immune system function. Comparative studies across different vector species have demonstrated that populations exposed to chronic temperature elevations exhibit enhanced heat tolerance, enabling them to survive in regions previously unsuitable for vector proliferation (Ferguson et al. 2010). One of the most critical physiological adaptations observed is the increase in desiccation resistance in arid and semiarid environments. Field-based studies on *A. aegypti* populations in regions experiencing prolonged droughts have revealed increased expression of cuticular hydrocarbons, which reduce water loss and enhance survival in hot, dry conditions. Similarly, laboratory experiments indicate that mosquitoes exposed to repeated heat stress develop higher metabolic efficiency, allowing them to sustain reproductive output under extreme temperatures. Beyond metabolic changes, immune responses of vectors are also being modulated by climate stress (Muturi et al. 2011). Elevated temperatures have been shown to enhance the replication rates of arboviruses within mosquitoes, thereby increasing vector competence. However, there is also evidence that chronic exposure to high temperatures can compromise mosquito immune defenses, making them more susceptible to certain pathogens. This paradoxical relationship between climate change, vector immunity, and pathogen development underscores the complex nature of climate-induced adaptations in disease vectors (Vasilakis and Gubler 2016).

A significant outcome of climate change is the rapid development of insecticide resistance among vector populations. Variations in temperature, precipitation patterns, and seasonal shifts not only influence the dynamics of vector populations but also trigger evolutionary processes that enhance resistance to chemical control methods. Research indicates that rising temperatures boost the activity of detoxification enzymes in mosquitoes, such as *Anopheles, Aedes*, and *Culex* (Abbasi and Daliri 2024a, 2024b, 2024c), which increases their resistance to commonly used insecticides. Additionally, higher temperatures contribute to behavioral modifications in vectors, reducing their exposure to insecticides. For example, *A. aegypti* mosquitoes in warmer regions tend to seek refuge in cooler indoor microhabitats, thereby decreasing the effectiveness of indoor residual spraying (IRS). Climate change may also facilitate gene flow among vector populations, promoting the spread of resistance alleles (Abbasi et al. 2025b). Studies show that resistant populations in tropical and subtropical regions, especially in Africa and Southeast Asia, have higher survival rates under thermal stress, which accelerates the natural selection process for resistance genes and their spread. In conclusion, climate change not only impacts vector survival directly but also influences insecticide resistance through physiological, behavioral, and genetic mechanisms. These changes present significant challenges to current vector control strategies and highlight the need for the development of climate-resilient approaches to counteract resistance evolution (Abbasi et al. 2022, 2024).

The transmission potential of VBDs is inherently linked to the EIP of pathogens, which determines how quickly an infected vector becomes capable of transmitting a pathogen. One of the most consistent findings across multiple studies is that rising temperatures accelerate the EIP of numerous pathogens, including *Plasmodium* spp, dengue virus, chikungunya virus, and West Nile virus (Alto and Bettinardi 2013). This results in shorter timeframes for vectors to become infectious, thereby increasing the frequency and intensity of disease outbreaks. For example, experimental studies on *A. aegypti* infected with dengue virus show that the virus reaches transmissible levels in the salivary glands more rapidly at 30–32°C compared to 25°C, thereby significantly enhancing transmission potential in warmer regions. Similarly, studies on *A. gambiae* indicate that malaria parasites complete their development twice as fast at 27°C compared to 20°C, resulting in a substantial increase in malaria transmission risk in tropical and subtropical areas undergoing warming trends (Blanford et al. 2013). Another key finding is that rising temperatures can alter vector–pathogen interactions at the molecular level. Transcriptomic analyses have shown that mosquitoes exposed to elevated temperatures exhibit differential expression of genes involved in antiviral responses and gut microbiome regulation, potentially influencing vector competence. The interaction between climate change, microbial symbionts, and vector immunity is an emerging area of research that requires further investigation to fully understand how these factors collectively shape disease transmission dynamics (LaDeau et al. 2015).

The microbiome of insect vectors significantly influences pathogen acquisition, replication, and transmission efficiency, and climate change—particularly rising temperatures—alters its composition and function, thereby affecting disease dynamics. Temperature fluctuations can shift the balance of symbiotic bacteria in mosquito guts, such as *Wolbachia* and *Asaia*, which interfere with pathogen replication. For example, elevated temperatures may increase *Wolbachia* density in *Aedes* mosquitoes, reducing their ability to transmit dengue and Zika viruses, whereas heat stress may suppress beneficial microbes, weakening their protective effects. Additionally, the microbiome interacts with the mosquito immune system, with high temperatures suppressing immune gene expression, leading to greater viral replication and higher transmission potential of arboviruses such as chikungunya and West Nile virus (Franklinos et al. 2019). Conversely, cooler temperatures may promote bacterial communities that enhance gut immunity and reduce infection susceptibility. Temperature changes also impact microbial metabolism, influencing pathogen viability through the production of reactive oxygen species (ROS) and other metabolites. While some bacteria produce ROS that suppress pathogens, extreme heat stress can disrupt these protective mechanisms, enhancing vector competence. Understanding the intricate relationship between temperature, microbiome composition, and pathogen transmission is critical for predicting climate-driven disease shifts. Future research should focus on microbiome-targeted interventions, such as probiotics or genetically engineered symbiotic bacteria, to mitigate the impact of climate change on vector competence (Tjaden et al. 2018).

Perhaps the most evident impact of climate change on insect vectors is the geographic expansion and seasonal extension of vector populations, leading to the emergence of diseases in previously unaffected regions. Multiple ecological modeling studies predict that warming temperatures will enable vector species to invade higher latitudes and altitudes, thereby increasing the risk of disease transmission in temperate regions (Negev et al. 2015). For instance, *Aedes albopictus*, a highly invasive mosquito species, has expanded its range northward into Europe and North America, facilitated by increasing temperatures and milder winters. Similarly, *I. scapularis*, the primary vector of Lyme disease, has been reported at higher latitudes in Canada and Scandinavia, where it was previously absent (Ogden et al. 2014). This geographic shift is not only driven by temperature increases but also by changes in precipitation patterns, which influence breeding site availability and larval survival rates. Beyond range expansion, climate change is also altering the seasonality of vector activity. Warmer temperatures are leading to earlier seasonal emergence and prolonged periods of vector activity, thereby extending transmission seasons for diseases such as dengue, malaria, and West Nile virus. In some regions, the traditional seasonality of VBDs is becoming less distinct, with outbreaks occurring unpredictably throughout the year. This shift presents significant challenges for vector surveillance and control programs, as traditional intervention strategies based on seasonal transmission patterns may no longer be effective (Rochlin et al. 2013).

**Fig. 1:**
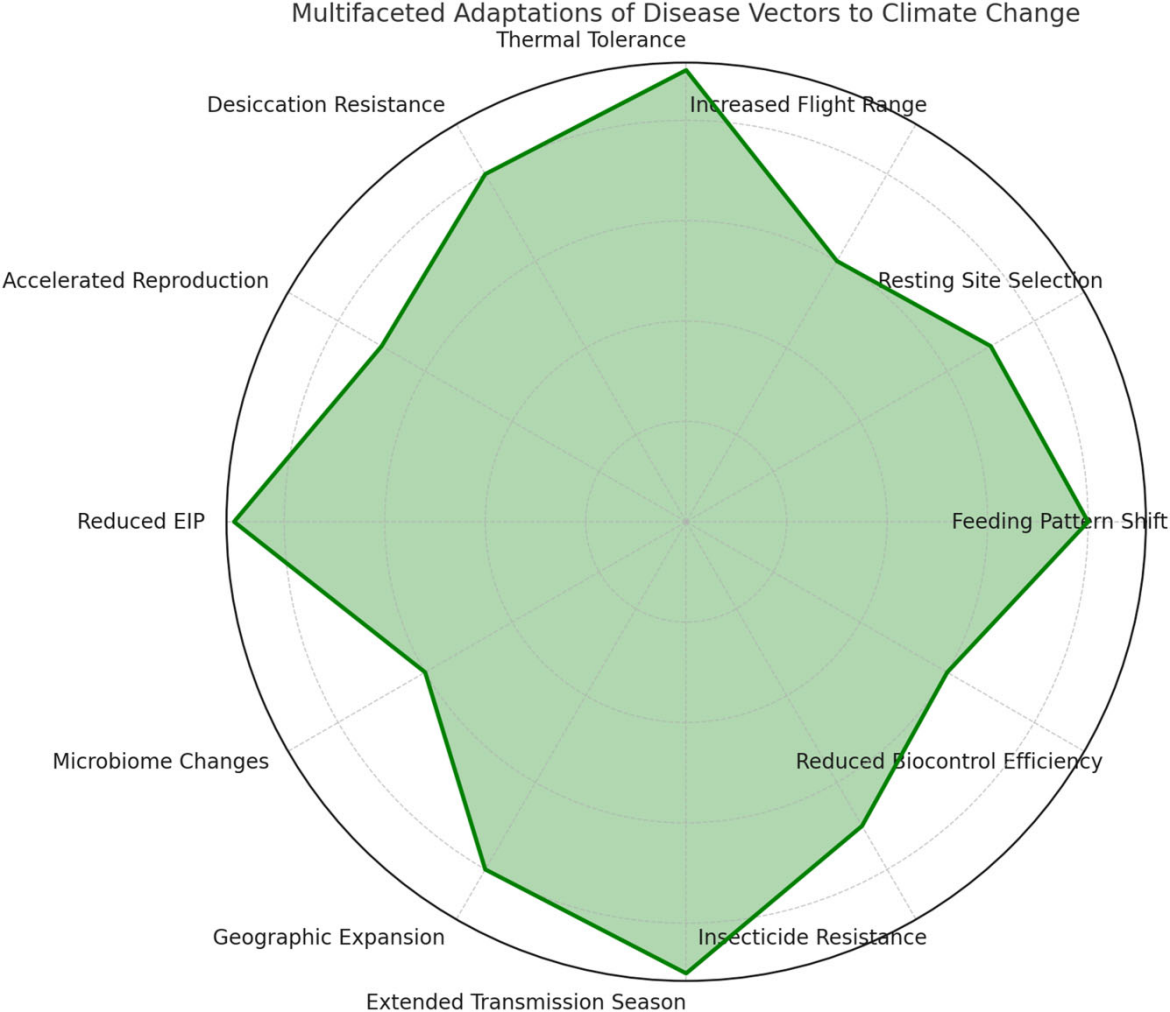
Hierarchical framework of climate change impacts on insect vectors: adaptations, epidemiological consequences, and control strategies.

The findings presented in this review highlight the urgent need for integrated vector management strategies that account for climate-induced behavioral and physiological adaptations. Traditional vector control measures, such as insecticide-treated nets (ITNs) and IRS, may become less effective as vectors modify their resting and feeding behaviors in response to climate stress (Ryan et al. 2019). Similarly, environmental management strategies must consider the changing distribution of breeding sites driven by altered precipitation patterns. Moreover, the increasing adaptability of vectors to climate stress underscores the need for real-time surveillance systems that incorporate climate data into vector risk assessments. Remote sensing technologies, coupled with climate-driven predictive modeling, could help identify emerging hotspots of disease transmission before outbreaks occur. Additionally, research on vector microbiomes and symbiotic bacteria, such as *Wolbachia*-based interventions, may offer novel strategies for mitigating vector competence under changing climatic conditions (Eisen and Moore 2013). In conclusion, the impact of climate change on vector behavior, physiology, and disease transmission is a complex and multifaceted phenomenon. The ability of insect vectors to adapt to climate-induced stressors will play a decisive role in shaping the future landscape of VBDs. Understanding these adaptive mechanisms is crucial for developing effective disease mitigation strategies, informing public health policies, and anticipating the emergence of novel disease threats in a rapidly changing world (Table 1 and Fig. 1) (Fischer et al. 2011).

**Table 1.** Summary of climate-driven adaptations in insect vectors and their epidemiological implications

**Adaptation type**	**Key observations**	**Mechanisms involved**	**Epidemiological implications**	**Challenges for vector control**	**Potential mitigation strategies**
**Behavioral adaptations**					
Shift in host-seeking behavior	Increased diurnal or crepuscular feeding patterns in *Aedes aegypti* and *Anopheles gambiae*	Thermal stress alters circadian rhythms; increased competition for nocturnal hosts	Higher human–vector contact rates, increased risk of arboviral and malarial transmission	Reduced efficacy of insecticide-treated bed nets (ITNs)	Development of daytime control measures such as repellents and targeted insecticide applications
Modification of resting site selection	Greater preference for indoor and shaded microhabitats	Behavioral thermoregulation; avoidance of extreme temperatures	Increased survival rates; higher resilience to temperature fluctuations	Reduced effectiveness of indoor residual spraying (IRS)	Integration of environmental management with IRS strategies
Expansion of flight range and dispersal patterns	Increased long-distance migration of *Aedes albopictus* in temperate zones	Enhanced flight endurance under warmer temperatures	Greater potential for disease spread to nonendemic regions	Increased difficulty in predicting outbreak locations	Climate-adaptive vector surveillance and remote sensing
**Physiological adaptations**					
Enhanced thermal tolerance	Increased survival of *Culex* and *Anopheles* mosquitoes at high temperatures	Upregulation of heat shock proteins; cuticular modifications	Expansion into hotter regions; prolonged seasonal activity	Conventional control measures designed for specific climatic ranges may fail	Targeted vector genomics to identify heat-resilient populations
Increased desiccation resistance	Prolonged survival in arid environments by *Aedes aegypti*	Cuticle thickening, altered lipid metabolism	Increased vector presence in dry regions; higher risk of urban dengue outbreaks	Traditional larval control strategies less effective	Use of bioengineered larvicides resistant to desiccation-prone larvae
Accelerated reproductive cycles	Shortened gonotrophic cycles in *Anopheles* spp under warming temperatures	Temperature-driven hormonal regulation	Faster population growth rates, increased disease transmission potential	Increased difficulty in reducing vector densities through conventional interventions	Use of reproductive inhibitors and genetic modification technologies
**Vector competence and pathogen dynamics**					
Reduced extrinsic incubation period (EIP)	Shorter EIP for malaria parasites, dengue virus, and Zika virus	Increased replication rates of pathogens in warmer vector bodies	Faster transmission cycles; higher epidemic potential	Increased frequency of outbreaks; reduced effectiveness of reactive control measures	Development of climate-resilient predictive models for epidemic forecasting
Changes in vector gut microbiome	Increased *Wolbachia*-mediated pathogen inhibition in mosquitoes	Temperature-induced shifts in microbial composition	Potential reduction in vector competence for some arboviruses	Unpredictable interactions between climate, microbiome, and vector–pathogen dynamics	Use of microbial-based vector control strategies
**Geographic and seasonal expansion**					
Poleward expansion of mosquito populations	*Aedes albopictus* now established in temperate zones	Milder winters enable overwintering of eggs and larvae	Emergence of arboviral diseases in nonendemic regions	Lack of immunity in newly affected populations	Early-warning systems and preemptive vector control measures
Longer transmission seasons	Extended activity of *Culex* spp and *Anopheles* spp	Delayed onset of winter conditions	Year-round disease transmission cycles	Inadequate seasonal vector control measures	Continuous monitoring and adaptive intervention programs
**Implications for vector control strategies**					
Reduced efficacy of insecticides	Increased resistance in *Anopheles* and *Aedes* mosquitoes	Enhanced detoxification enzyme expression	Higher survival rates of resistant vector populations	Need for alternative control methods	The increasing frequency of insecticide resistance necessitates the integration of alternative control strategies, such as genetic modifications, *Wolbachia*-based interventions, and climate-adaptive insecticide rotation programs
Declining effectiveness of biological control methods	Altered predator–prey dynamics in response to climate change	Reduced efficacy of natural mosquito predators in some habitats	Potential breakdown of ecosystem-based vector control approaches	Need for artificial biological control reinforcements	

## Discussion

This comprehensive review highlights the profound influence of climate change on the behavioral and physiological adaptations of insect vectors, emphasizing its cascading effects on VBD transmission dynamics. The findings reveal that temperature-driven behavioral modifications, enhanced physiological plasticity, altered vector competence, and geographic redistribution of vector populations are collectively reshaping the epidemiology of several major VBDs (Rocklöv et al. 2016). These climate-induced adaptations necessitate a reevaluation of current vector control strategies and public health interventions. This section critically examines the implications of the observed findings, identifies existing research gaps, and discusses potential future directions for mitigating the impacts of climate-driven vector adaptations on disease transmission. The findings of this review demonstrate that insect vectors, particularly mosquitoes and ticks, exhibit substantial behavioral plasticity in response to rising temperatures. One of the most critical shifts observed is the modification of host-seeking and biting patterns, which directly affects the frequency and intensity of human–vector interactions. Traditionally, mosquito species such as *A. gambiae* and *A. aegypti* were believed to exhibit strict nocturnal biting behavior; however, recent evidence suggests that warming temperatures are causing a shift toward diurnal or crepuscular feeding habits (Rocklöv et al. 2016). This behavioral modification significantly undermines the efficacy of conventional vector control measures such as insecticide-treated bed nets (ITNs), which are primarily designed for nighttime protection. Another key behavioral adaptation involves changes in resting site selection, with mosquitoes increasingly seeking cooler microhabitats in response to thermal stress. This shift not only enhances their survival in extreme climatic conditions but also affects their exposure to IRS interventions (Siiba et al. 2024). Furthermore, host preference modifications, particularly the observed increase in anthropophily (preference for human hosts) in certain mosquito species, have critical epidemiological implications. A higher affinity for human hosts accelerates disease transmission by increasing the frequency of vector–human contact, thereby shortening the interval between successive pathogen transmission events. These behavioral adaptations collectively contribute to increased disease transmission efficiency, as vectors become more resilient to climate-induced environmental changes. Consequently, traditional epidemiological models that assume static vector behavior must be updated to incorporate the dynamically shifting feeding, resting, and host-seeking behaviors of insect vectors in response to climate change (Takken and Verhulst 2013).

Southeast Asia has experienced notable shifts in vector behavior that have challenged malaria and dengue control efforts. In Thailand, *A. aegypti* mosquitoes have altered their peak biting times from early morning and late afternoon to nighttime, diminishing the effectiveness of daytime vector control strategies. Similarly, in Indonesia, *Anopheles* mosquitoes increasingly rest outdoors rather than indoors, reducing the impact of insecticide-treated bed nets (ITNs) and IRS (Abbasi 2025e). These behavioral adaptations necessitate innovative control measures, such as targeted outdoor spraying and the release of *Wolbachia*-infected mosquitoes to reduce vector competence (Abbasi and Moemenbellah-Fard 2024). In response, human adaptation strategies have emerged to mitigate disease risks. In malaria-endemic regions, communities have modified housing structures by incorporating insect-proof netting and improved ventilation, while urban areas prone to dengue have implemented water storage management and community-based vector control initiatives (Abedi-Astaneh et al. 2025). Additionally, integrating climate-informed disease forecasting into public health planning facilitates early interventions and outbreak preparedness. These adaptive approaches, alongside conventional control methods, underscore the need for interdisciplinary strategies to combat climate-driven VBD transmission (Smith et al. 2012).

Physiological plasticity has emerged as a critical determinant of vector adaptability under climate change. The ability of insect vectors to adjust their thermal tolerance, metabolism, and immune responses plays a pivotal role in their survival and reproductive success. The review findings indicate that increased heat tolerance and desiccation resistance are among the most common physiological adaptations observed in mosquito populations exposed to prolonged temperature elevations (malERA Refresh Consultative Panel on Combination Interventions and Modelling 2017). Several studies have reported that higher temperatures induce cuticular modifications in mosquitoes, leading to reduced water loss and enhanced desiccation resistance. This adaptation enables vectors to survive in arid and semiarid environments that were previously unsuitable for their proliferation. Additionally, thermal stress-induced metabolic adjustments, including enhanced lipid metabolism and upregulated heat shock protein expression, allow vectors to sustain reproductive output even under extreme conditions (King et al. 2011). Perhaps one of the most significant implications of physiological plasticity is its role in vector longevity and reproductive success. Warmer temperatures have been shown to accelerate gonotrophic cycles (the time interval between blood-feeding and egg-laying in female mosquitoes), leading to higher vector densities and increased disease transmission potential. The observed increase in multiple blood-feeding behavior under elevated temperatures further amplifies this effect, as vectors ingest and transmit pathogens more frequently. From a control perspective, these physiological adaptations pose a formidable challenge to existing intervention strategies (Murdock et al. 2013). The increased survival rates and prolonged vector activity reduce the efficacy of traditional vector control tools such as chemical insecticides, as longer-lived vectors are more likely to develop resistance. Moreover, the heightened reproductive potential of climate-adapted vectors underscores the urgent need for novel vector control approaches, including genetic and microbiome-based interventions (Raliya 2019).

Traditional vector control methods, such as ITNs, IRS, and larval source management, have been essential for preventing VBDs. However, climate change has introduced new challenges that diminish the effectiveness of these approaches. Rising temperatures, altered precipitation patterns, and prolonged transmission seasons have affected vector behavior, physiology, and distribution, leading to reduced efficacy of conventional methods. Increased resistance to insecticides, accelerated by climate-driven selection pressures, has compromised ITNs and IRS. Additionally, mosquitoes are exhibiting altered feeding patterns and resting behaviors, reducing their exposure to insecticide-treated surfaces, and climate change is expanding vector populations into new habitats, especially in temperate regions (Abbasi 2025b, 2025a). In response, climate-adapted control strategies are being developed, including genetic approaches like gene drive technologies to suppress vector populations, microbiome-based interventions such as using *Wolbachia* bacteria to control virus replication in mosquitoes, and climate-informed surveillance systems (Abbasi et al. 2025a). Integrated vector management combining chemical, biological, and environmental strategies is essential for long-term control under climate variability. The development of climate-resilient control measures, enhanced global surveillance, and the use of climate data in risk assessments are crucial to managing future VBD outbreaks (Abbasi and Saeedi 2022; Talbalaghi et al. 2024).

One of the most concerning outcomes of climate-driven vector adaptations is the observed increase in vector competence—the ability of a vector to acquire, harbor, and transmit pathogens. This review highlights that rising temperatures shorten the EIP of several major pathogens, including *Plasmodium* spp, dengue virus, chikungunya virus, and West Nile virus. A reduced EIP allows infected vectors to transmit pathogens more quickly, leading to higher transmission rates and increased outbreak frequency. Molecular studies suggest that temperature-induced changes in vector gut microbiota and immune responses may further enhance vector competence. For instance, elevated temperatures have been linked to upregulated expression of viral replication genes in *A. aegypti*, leading to increased viral loads and heightened transmission efficiency (Alto and Bettinardi 2013; Gao et al. 2020). Similarly, transcriptomic analyses indicate that thermal stress alters the expression of genes involved in pathogen recognition and immune modulation, potentially compromising the vector's ability to mount an effective immune response against invading pathogens. The impact of climate change on vector competence also extends to secondary vector species, which may previously have played a minor role in disease transmission. Warming temperatures allow these secondary vectors to become more competent disease carriers, expanding the range of transmission networks and increasing the complexity of disease epidemiology. This phenomenon has already been observed in the emergence of dengue and chikungunya in temperate regions, where previously nonendemic mosquito species have become efficient vectors under favorable climatic conditions (Mordecai et al. 2017; Tesla et al. 2018).

Climate change is fundamentally reshaping the spatial and temporal distribution of insect vectors, leading to the emergence of VBDs in regions previously considered nonendemic. Multiple predictive models suggest that rising temperatures and changing precipitation patterns are facilitating the poleward expansion of mosquito and tick populations, bringing diseases such as malaria, dengue, and Lyme disease to new geographic areas (Caminade et al. 2019). For instance, the northward expansion of *A. albopictus* in Europe and North America has been documented in response to warming temperatures and milder winters. Similarly, the observed increase in tick-borne disease incidence in northern latitudes, including Lyme disease and tick-borne encephalitis, reflects the successful adaptation of vector populations to extended transmission seasons (Ogden et al. 2014). These shifts have profound public health implications, as regions with limited immunity and inadequate health care infrastructure become increasingly vulnerable to VBD outbreaks. Beyond geographic expansion, climate change is also driving longer transmission seasons, as higher temperatures allow vectors to remain active for extended periods. This shift disrupts traditional seasonal patterns of disease transmission, making outbreaks less predictable and more difficult to control. Consequently, public health strategies must transition from seasonal intervention models to continuous surveillance and adaptive response mechanisms to mitigate the risks posed by prolonged vector activity (Couper et al. 2021).

Climate change imposes selective pressures on insect vectors, driving both behavioral and physiological adaptations that enhance their survival and transmission potential. These adaptations are interconnected, as physiological changes often support or reinforce behavioral shifts. Rising temperatures increase metabolic rates in mosquitoes, leading to higher energy demands and altered feeding behaviors (Caminade et al. 2019; Abbasi 2025c). For example, *A. aegypti* exposed to elevated temperatures shows increased biting frequency, particularly during the day, which reduces the effectiveness of nighttime interventions such as ITNs. Additionally, changes in host-seeking behavior are linked to physiological stress responses, with mosquitoes shifting from animal-preference to human-preference feeding patterns, likely due to thermal stress-induced alterations in olfactory receptor sensitivity. This shift, combined with heightened reproductive demands, reinforces human–vector contact rates. Furthermore, temperature fluctuations affect vector immune responses, with chronic heat stress suppressing immune function and increasing pathogen transmission rates, particularly for arboviruses such as dengue and chikungunya. Climate change also enhances desiccation resistance in mosquitoes and ticks through increased cuticular lipid composition, allowing them to persist in drier environments and altering traditional disease transmission dynamics (Abbasi 2025d; Abbasi et al. 2025c). The interplay between these behavioral and physiological adaptations underscores the complexity of climate-driven vector responses. Future research should explore the molecular and genetic mechanisms underlying these changes to develop targeted control strategies that account for both behavioral and physiological resilience (Ma et al. 2022).

The findings of this review underscore the urgent need for integrated vector management approaches that account for climate-induced behavioral and physiological adaptations. Traditional control measures, including insecticide-based interventions, environmental management, and bed nets, may become increasingly ineffective as vectors modify their behavior and physiology (Achee et al. 2015; Carvalho et al. 2015). Emerging alternative strategies include, such as gene drive technologies targeting vector reproductive capacity (Genetic engineering approaches), including the use of *Wolbachia* bacteria to reduce vector competence (microbiome-based interventions), integrating remote sensing and predictive modeling to anticipate vector range expansions and outbreak risks (climate-informed vector surveillance systems) (Flores and O'Neill 2018). Future research must also focus on the complex interplay between climate change, vector genetics, and pathogen evolution, as the rapid adaptation of vectors may accelerate the emergence of novel disease variants. Additionally, transdisciplinary collaborations between entomologists, climate scientists, epidemiologists, and public health policymakers will be essential in developing holistic strategies for mitigating the impact of climate-driven vector adaptations (Nagi 2023).

## Conclusion

In conclusion, climate change is not only reshaping vector behavior and physiology but also fundamentally altering the global epidemiology of VBDs. Understanding and anticipating these adaptations is critical for developing resilient public health interventions, ensuring that disease control strategies remain effective in an era of rapidly changing environmental conditions. Future research should prioritize genomic studies to uncover the molecular mechanisms underlying vector adaptation, as well as long-term surveillance programs to track evolving transmission dynamics and inform targeted intervention strategies (Caminade et al. 2014; Ogden and Lindsay 2016).

## Supplementary Material

obaf011_Supplemental_Files

## Data Availability

All data generated or analyzed during this study are included in this published article.
