# Supplementary material for: Climate Change and Vector-Borne Disease Transmission: The Role of Insect Behavioral and Physiological Adaptations
Source: Integr Org Biol. 2025 Mar 19;7(1):obaf011. doi: 10.1093/iob/obaf011 (PMC12053451; doi:10.1093/iob/obaf011)
Supplement: obaf011_Supplemental_Files [file obaf011_supplemental_files.zip › Abstract translations.docx]

**Abstract**

Climate change is profoundly reshaping the behavior, physiology, and distribution of insect vectors, with significant implications for vector-borne disease transmission. Rising temperatures, shifting precipitation patterns, and extreme weather events are driving behavioral adaptations such as altered host-seeking patterns, modified resting site preferences, and extended seasonal activity. Concurrently, vectors exhibit physiological plasticity, including enhanced thermal tolerance, desiccation resistance, and accelerated reproductive cycles, which contribute to increased survival and vector competence. This review synthesizes current research on climate-driven adaptations in major disease vectors, focusing on their epidemiological consequences and implications for public health interventions. A systematic literature review was conducted using major scientific databases to assess the impact of climate change on insect vector adaptation. Studies examining temperature-induced behavioral shifts, physiological modifications, and changes in vector competence were analyzed to identify emerging trends and knowledge gaps. Findings indicate that climate-driven vector adaptations are increasing the efficiency of disease transmission, enabling the geographic expansion of vector populations and prolonging transmission seasons. These changes challenge existing vector control strategies, necessitating innovative approaches such as genetic engineering, microbiome-based interventions, and climate-informed surveillance systems. Given the accelerating impact of climate change, there is an urgent need for adaptive, evidence-based control strategies to mitigate the growing threat of vector-borne diseases and enhance global health resilience

**Keywords:** Climate change, insect vectors, vector adaptation, behavioral shifts, physiological plasticity, vector competence, vector-borne diseases

**Zusammenfassung (German)**

Der Klimawandel verändert tiefgreifend das Verhalten, die Physiologie und die Verbreitung von Insektenvektoren, was erhebliche Auswirkungen auf die Übertragung vektorübertragener Krankheiten hat. Steigende Temperaturen, veränderte Niederschlagsmuster und extreme Wetterereignisse führen zu Verhaltensanpassungen wie veränderten Wirtsuchmustern, modifizierten Ruheplatzpräferenzen und verlängerter saisonaler Aktivität. Gleichzeitig zeigen Vektoren physiologische Plastizität, darunter eine erhöhte Hitzetoleranz, Resistenz gegen Austrocknung und beschleunigte Fortpflanzungszyklen, was zu einer höheren Überlebensrate und Vektorkompetenz beiträgt.
Diese Übersichtsarbeit fasst aktuelle Forschungsergebnisse zu klimabedingten Anpassungen wichtiger Krankheitsvektoren zusammen, mit Schwerpunkt auf ihren epidemiologischen Folgen und Implikationen für öffentliche Gesundheitsmaßnahmen. Eine systematische Literaturrecherche wurde in führenden wissenschaftlichen Datenbanken durchgeführt, um die Auswirkungen des Klimawandels auf die Anpassung von Insektenvektoren zu bewerten. Studien, die temperaturinduzierte Verhaltensänderungen, physiologische Modifikationen und Veränderungen der Vektorkompetenz untersuchten, wurden analysiert, um aufkommende Trends und Forschungslücken zu identifizieren.
Die Ergebnisse zeigen, dass klimabedingte Anpassungen von Vektoren die Effizienz der Krankheitsübertragung erhöhen, die geografische Ausbreitung von Vektorpopulationen begünstigen und die Übertragungszeiten verlängern. Diese Veränderungen stellen bestehende Vektorkontrollstrategien vor große Herausforderungen und erfordern innovative Ansätze wie genetische Modifikationen, mikrobiombasierte Interventionen und klimainformierte Überwachungssysteme. Angesichts der zunehmenden Auswirkungen des Klimawandels besteht ein dringender Bedarf an adaptiven, evidenzbasierten Kontrollstrategien, um die wachsende Bedrohung durch vektorübertragene Krankheiten zu mindern und die globale Gesundheitsresilienz zu stärken.

**Schlüsselwörter:** Klimawandel, Insektenvektoren, Vektoranpassung, Verhaltensänderungen, physiologische Plastizität, Vektorkompetenz, vektorübertragene Krankheiten

**Resumen (Spanish)**

El cambio climático está remodelando profundamente el comportamiento, la fisiología y la distribución de los insectos vectores, con importantes implicaciones para la transmisión de enfermedades transmitidas por vectores. El aumento de las temperaturas, los cambios en los patrones de precipitación y los eventos climáticos extremos están impulsando adaptaciones conductuales, como modificaciones en la búsqueda de hospedadores, cambios en las preferencias de los sitios de reposo y una actividad estacional prolongada. Paralelamente, los vectores exhiben plasticidad fisiológica, incluyendo una mayor tolerancia térmica, resistencia a la desecación y ciclos reproductivos acelerados, lo que contribuye a una mayor supervivencia y competencia vectorial. Esta revisión sintetiza la investigación actual sobre las adaptaciones impulsadas por el clima en los principales vectores de enfermedades, centrándose en sus consecuencias epidemiológicas y sus implicaciones para las intervenciones en salud pública. Se realizó una revisión sistemática de la literatura utilizando las principales bases de datos científicas para evaluar el impacto del cambio climático en la adaptación de los insectos vectores. Se analizaron estudios que examinan cambios conductuales inducidos por la temperatura, modificaciones fisiológicas y alteraciones en la competencia vectorial con el fin de identificar tendencias emergentes y lagunas de conocimiento. Los hallazgos indican que las adaptaciones de los vectores impulsadas por el cambio climático están aumentando la eficiencia de la transmisión de enfermedades, permitiendo la expansión geográfica de las poblaciones de vectores y prolongando las temporadas de transmisión. Estos cambios desafían las estrategias de control de vectores existentes, lo que hace necesaria la adopción de enfoques innovadores, como la ingeniería genética, intervenciones basadas en el microbioma y sistemas de vigilancia informados por el clima. Dado el impacto acelerado del cambio climático, es urgente desarrollar estrategias de control adaptativas y basadas en evidencia para mitigar la creciente amenaza de las enfermedades transmitidas por vectores y fortalecer la resiliencia sanitaria global.

**Palabras clave**: Cambio climático, insectos vectores, adaptación de vectores, cambios conductuales, plasticidad fisiológica, competencia vectorial, enfermedades transmitidas por vectores.

**Abstract (Italian)**

Il cambiamento climatico sta rimodellando profondamente il comportamento, la fisiologia e la distribuzione degli insetti vettori, con importanti implicazioni per la trasmissione delle malattie trasmesse da vettori. L’aumento delle temperature, le variazioni nei modelli di precipitazione e gli eventi meteorologici estremi stanno determinando adattamenti comportamentali, come cambiamenti nei modelli di ricerca dell’ospite, modifiche nelle preferenze dei siti di riposo e un’attività stagionale prolungata. Contemporaneamente, i vettori mostrano una plasticità fisiologica, tra cui una maggiore tolleranza termica, resistenza alla disidratazione e cicli riproduttivi accelerati, contribuendo a una maggiore sopravvivenza e competenza vettoriale. Questa revisione sintetizza le ricerche attuali sulle adattamenti dei principali vettori di malattie indotte dal cambiamento climatico, concentrandosi sulle conseguenze epidemiologiche e sulle implicazioni per le strategie di sanità pubblica. È stata condotta una revisione sistematica della letteratura utilizzando le principali banche dati scientifiche per valutare l'impatto del cambiamento climatico sull’adattamento degli insetti vettori. Sono stati analizzati studi che esaminano le variazioni comportamentali indotte dalla temperatura, le modificazioni fisiologiche e i cambiamenti nella competenza vettoriale al fine di identificare tendenze emergenti e lacune di conoscenza. I risultati indicano che gli adattamenti dei vettori determinati dal cambiamento climatico stanno aumentando l’efficienza della trasmissione delle malattie, consentendo l’espansione geografica delle popolazioni vettoriali e prolungando le stagioni di trasmissione. Questi cambiamenti sfidano le attuali strategie di controllo dei vettori, rendendo necessaria l’adozione di approcci innovativi come l’ingegneria genetica, le interventi basati sul microbioma e i sistemi di sorveglianza informati dal clima. Considerando l’impatto accelerato del cambiamento climatico, è urgente sviluppare strategie di controllo adattive e basate sull’evidenza per mitigare la crescente minaccia delle malattie trasmesse da vettori e rafforzare la resilienza sanitaria globale.

**Parole chiave**: Cambiamento climatico, insetti vettori, adattamento dei vettori, cambiamenti comportamentali, plasticità fisiologica, competenza vettoriale, malattie trasmesse da vettori.

**الملخص (Arabic)**

يُعيد التغير المناخي تشكيل سلوك الحشرات الناقلة للأمراض وفسيولوجيتها وتوزيعها بشكل عميق، مما يترتب عليه آثار كبيرة على انتقال الأمراض المنقولة بالنواقل. تؤدي ارتفاع درجات الحرارة، وتغير أنماط الهطول، والظواهر الجوية المتطرفة إلى تغييرات سلوكية مثل تعديل أنماط البحث عن العائل، وتفضيلات مواقع الاستراحة، وزيادة النشاط الموسمي. بالتزامن مع ذلك، تظهر النواقل مرونة فسيولوجية تشمل زيادة تحمل الحرارة، ومقاومة الجفاف، وتسريع دورات التكاثر، مما يسهم في زيادة معدلات البقاء والكفاءة الناقلة. تستعرض هذه المراجعة الأبحاث الحالية حول التكيفات التي تحركها التغيرات المناخية في النواقل الرئيسية للأمراض، مع التركيز على العواقب الوبائية والتداعيات على التدخلات الصحية العامة. تم إجراء مراجعة منهجية للأدبيات العلمية باستخدام قواعد البيانات العلمية الكبرى لتقييم تأثير التغير المناخي على تكيف الحشرات الناقلة للأمراض. تم تحليل الدراسات التي تفحص التغيرات السلوكية الناجمة عن درجة الحرارة، والتعديلات الفسيولوجية، والتغيرات في كفاءة النقل، بهدف تحديد الاتجاهات الناشئة والفجوات المعرفية. تشير النتائج إلى أن التكيفات المناخية للنواقل تزيد من كفاءة انتقال الأمراض، مما يسمح بالتوسع الجغرافي لمجموعات النواقل وإطالة مواسم الانتقال. تشكل هذه التغيرات تحديًا لاستراتيجيات مكافحة النواقل الحالية، مما يستلزم اعتماد أساليب مبتكرة مثل الهندسة الوراثية، والتدخلات القائمة على الميكروبيوم، وأنظمة المراقبة المستندة إلى المناخ. نظرًا للتأثير المتسارع للتغير المناخي، هناك حاجة ملحة إلى استراتيجيات تكيفية قائمة على الأدلة للحد من التهديد المتزايد للأمراض المنقولة بالنواقل وتعزيز مرونة الصحة العالمية.

**الكلمات المفتاحية**: التغير المناخي، الحشرات الناقلة، تكيف النواقل، التغيرات السلوكية، المرونة الفسيولوجية، كفاءة النقل، الأمراض المنقولة بالنواقل.

**چکیده (Persian)**

تغییرات اقلیمی به‌طور چشمگیری رفتار، فیزیولوژی و پراکنش ناقلین حشرات را دگرگون کرده و پیامدهای مهمی برای انتقال بیماری‌های منتقله توسط ناقلین به همراه دارد. افزایش دما، تغییر الگوهای بارندگی و رخدادهای شدید جوی باعث سازگاری‌های رفتاری مانند تغییر در الگوهای جستجوی میزبان، اصلاح ترجیحات زیستگاه‌های استراحت و افزایش فعالیت فصلی ناقلین شده است. هم‌زمان، ناقلین انعطاف‌پذیری فیزیولوژیکی قابل‌توجهی از خود نشان می‌دهند، از جمله افزایش تحمل حرارتی، مقاومت در برابر خشکی و چرخه‌های تولیدمثلی سریع‌تر که به بقای بیشتر و افزایش کارایی آن‌ها در انتقال بیماری‌ها کمک می‌کند. این مطالعه مروری به بررسی تحقیقات اخیر در مورد سازگاری‌های ناقلین اصلی بیماری‌ها در پاسخ به تغییرات اقلیمی پرداخته و پیامدهای اپیدمیولوژیک و مداخلات سلامت عمومی مرتبط را مورد تحلیل قرار داده است.

یک مرور سیستماتیک از مطالعات منتشرشده در پایگاه‌های داده علمی معتبر برای ارزیابی اثر تغییرات اقلیمی بر سازگاری ناقلین حشرات انجام شد. در این مطالعه، تحقیقات مرتبط با تغییرات رفتاری ناشی از دما، اصلاحات فیزیولوژیکی و تغییرات در کارایی انتقال بیماری توسط ناقلین مورد تجزیه‌وتحلیل قرار گرفت تا روندهای نوظهور و شکاف‌های دانشی شناسایی شوند. یافته‌ها نشان می‌دهند که سازگاری‌های اقلیمی ناقلین باعث افزایش کارایی انتقال بیماری، گسترش جغرافیایی جمعیت‌های ناقل و طولانی‌تر شدن فصول انتقال بیماری می‌شود. این تغییرات راهبردهای کنونی کنترل ناقلین را به چالش کشیده و نیازمند به‌کارگیری رویکردهای نوآورانه‌ای مانند مهندسی ژنتیک، مداخلات مبتنی بر میکروبیوم و سیستم‌های پایش مبتنی بر تغییرات اقلیمی است. با توجه به تأثیرات فزاینده تغییرات اقلیمی، تدوین راهبردهای کنترلی سازگار و مبتنی بر شواهد برای کاهش تهدید روزافزون بیماری‌های منتقله توسط ناقلین و تقویت تاب‌آوری جهانی در برابر این بیماری‌ها ضروری است.

**واژگان کلیدی**: تغییرات اقلیمی، ناقلین حشرات، سازگاری ناقلین، تغییرات رفتاری، انعطاف‌پذیری فیزیولوژیکی، کارایی انتقال بیماری، بیماری‌های منتقله توسط ناقلین.

**Résumé (French)**

Le changement climatique remodèle profondément le comportement, la physiologie et la distribution des insectes vecteurs, avec des implications majeures pour la transmission des maladies à transmission vectorielle. L’augmentation des températures, les modifications des régimes de précipitations et les phénomènes météorologiques extrêmes entraînent des adaptations comportementales telles que des changements dans les schémas de recherche d’hôtes, des modifications des préférences en matière de sites de repos et une activité saisonnière prolongée. Parallèlement, les vecteurs présentent une plasticité physiologique, notamment une tolérance thermique accrue, une résistance à la dessiccation et des cycles de reproduction accélérés, contribuant ainsi à une plus grande survie et à une compétence vectorielle améliorée. Cette revue synthétise les recherches actuelles sur les adaptations des principaux vecteurs de maladies induites par le climat, en mettant l’accent sur leurs conséquences épidémiologiques et leurs implications pour les interventions en santé publique. Une revue systématique de la littérature a été réalisée à l’aide des principales bases de données scientifiques afin d’évaluer l’impact du changement climatique sur l’adaptation des insectes vecteurs. Des études examinant les modifications comportementales induites par la température, les changements physiologiques et l’évolution de la compétence vectorielle ont été analysées afin d’identifier les tendances émergentes et les lacunes dans les connaissances. Les résultats indiquent que les adaptations des vecteurs liées au climat augmentent l’efficacité de la transmission des maladies, permettant l’expansion géographique des populations de vecteurs et prolongeant les saisons de transmission. Ces changements remettent en question les stratégies actuelles de lutte antivectorielle, nécessitant l’adoption d’approches innovantes telles que l’ingénierie génétique, les interventions basées sur le microbiome et les systèmes de surveillance informés par le climat. Face à l’impact croissant du changement climatique, il est urgent de développer des stratégies de contrôle adaptatives et fondées sur des preuves afin d’atténuer la menace croissante des maladies vectorielles et de renforcer la résilience sanitaire mondiale.

**Mots-clés** : Changement climatique, insectes vecteurs, adaptation des vecteurs, modifications comportementales, plasticité physiologique, compétence vectorielle, maladies à transmission vectorielle.

摘要 (Chinese)

气候变化正在深刻影响昆虫媒介的行为、生理特性和分布模式，对媒介传播疾病的传播产生重大影响。气温上升、降水模式变化及极端天气事件正在驱动媒介的行为适应，例如改变寻宿主模式、调整栖息地偏好以及延长季节性活动。同时，媒介表现出生理可塑性，包括增强的耐热性、抗干旱能力及加速的繁殖周期，这些因素共同提高了其生存能力和媒介适能。本综述综合了当前关于气候驱动的主要疾病媒介适应性的研究，重点探讨其流行病学后果及对公共卫生干预的影响。通过系统性文献回顾，我们利用主要科学数据库评估了气候变化对昆虫媒介适应性的影响，分析了有关温度诱导的行为变化、生理调节及媒介适能变化的研究，以识别新兴趋势和知识空白。研究结果表明，气候驱动的媒介适应性正在提高疾病传播效率，促进媒介种群的地理扩张，并延长传播季节。这些变化对现有媒介控制策略提出挑战，亟需采用创新方法，如基因工程、基于微生物组的干预措施及气候驱动的监测系统。鉴于气候变化影响的加速，迫切需要基于科学证据的适应性控制策略，以缓解媒介传播疾病日益增长的威胁，并增强全球卫生韧性。

**关键词**：气候变化、昆虫媒介、媒介适应、行为变化、生理可塑性、媒介适能、媒介传播疾病

**Аннотация (Russian)**

Изменение климата существенно трансформирует поведение, физиологию и распространение насекомых-векторов, что имеет значительные последствия для передачи заболеваний, передаваемых через векторы. Повышение температур, изменения в режимах осадков и экстремальные погодные явления способствуют поведенческой адаптации, включая изменение стратегий поиска хозяина, предпочтений в местах отдыха и удлинение сезонов активности. Одновременно векторы демонстрируют физиологическую пластичность, такую как повышенная термостойкость, устойчивость к высыханию и ускоренные репродуктивные циклы, что способствует их выживанию и увеличению способности передавать патогены. В данном обзоре обобщены современные исследования, посвященные адаптациям основных векторов заболеваний под воздействием климатических факторов, с акцентом на эпидемиологические последствия и влияние на меры общественного здравоохранения.

Был проведён систематический обзор литературы с использованием ведущих научных баз данных для оценки влияния изменения климата на адаптацию насекомых-векторов. Были проанализированы исследования, рассматривающие поведенческие изменения, вызванные температурными колебаниями, физиологические модификации и изменения в способности передавать патогены, с целью выявления новых тенденций и пробелов в знаниях. Полученные данные свидетельствуют о том, что климатически обусловленные адаптации векторов увеличивают эффективность передачи заболеваний, способствуют расширению ареала популяций векторов и продлевают сезоны передачи инфекций. Эти изменения создают вызовы для существующих стратегий борьбы с векторами, требуя внедрения инновационных подходов, таких как генная инженерия, методы, основанные на микробиоме, и системы климатически информированного эпиднадзора. С учётом ускоряющегося воздействия изменения климата, необходимо срочное внедрение адаптивных и научно обоснованных стратегий контроля для снижения растущей угрозы заболеваний, передаваемых через векторы, и укрепления глобальной устойчивости здравоохранения.

**Ключевые слова**: изменение климата, насекомые-векторы, адаптация векторов, поведенческие изменения, физиологическая пластичность, способность векторов к передаче заболеваний, заболевания, передаваемые через векторы.
