# Supplementary figures and images for: Climate Change and Vector-Borne Disease Transmission: The Role of Insect Behavioral and Physiological Adaptations
Source: Integr Org Biol. 2025 Mar 19;7(1):obaf011. doi: 10.1093/iob/obaf011 (PMC12053451; doi:10.1093/iob/obaf011)

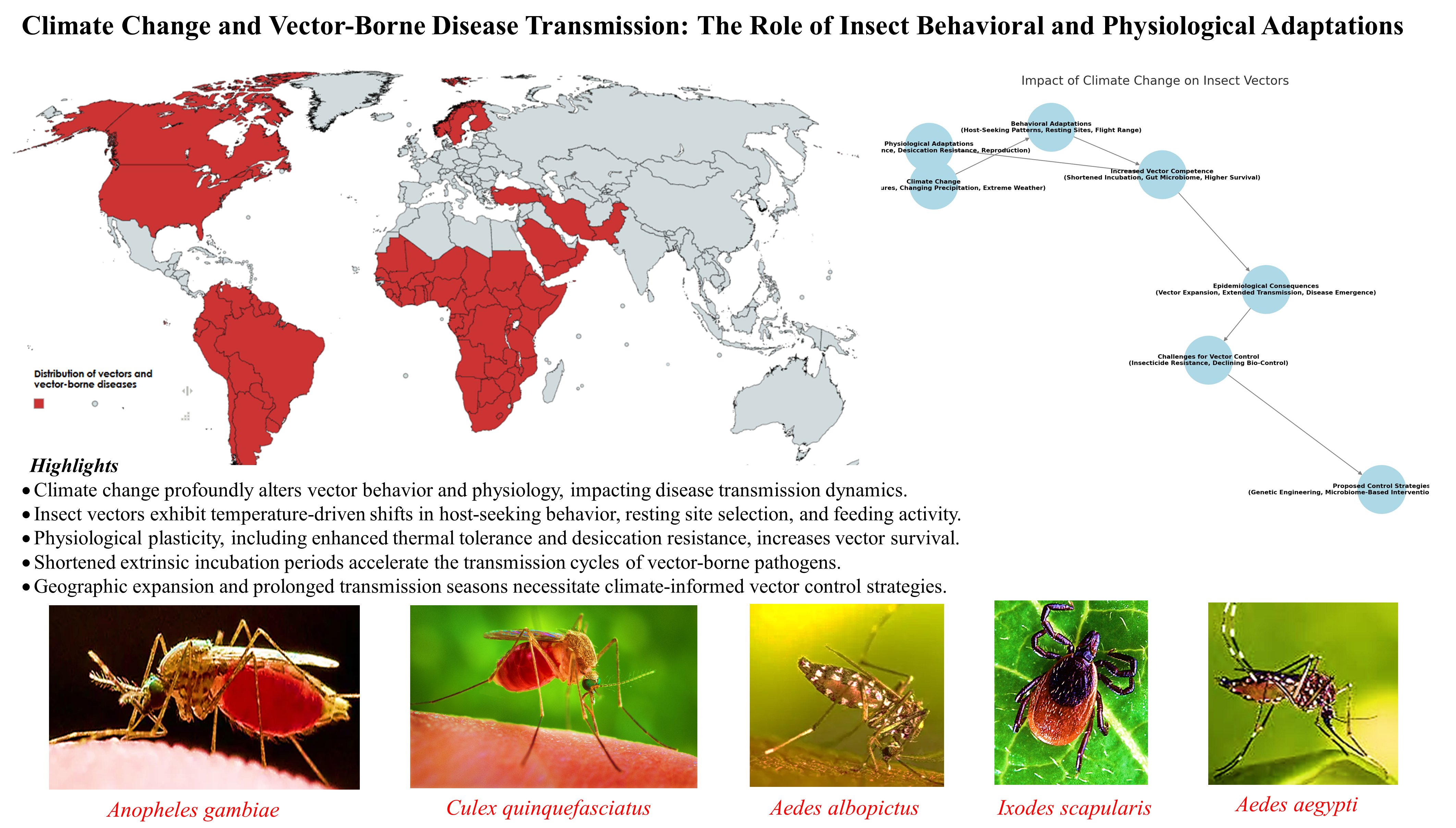

Supplement: obaf011_Supplemental_Files [file obaf011_supplemental_files.zip › Graphical Abstract.tif]
